# A Cooperative Routing Scheme Using Inter-Satellite Links to Assist Data Downloading for LEO Satellite Networks †

**DOI:** 10.3390/s22207986

**Published:** 2022-10-19

**Authors:** Zijian Yang, Huijie Liu, Jifeng Jin, Feng Tian

**Affiliations:** 1 Innovation Academy for Microsatellites of CAS, Shanghai 201203, China; 2 University of Chinese Academy of Sciences, Beijing 100044, China

**Keywords:** LEO satellite systems, network flow, satellite data downloading, routing algorithm

## Abstract

Low earth orbit (LEO) satellite networks can provide Internet service to users in areas where cellular networks are difficult to deploy. One critical function of satellites is to transfer data from satellite networks to ground core network through earth stations (ESs). The Ka-band multiple-input multiple-output (MIMO) can be used to establish feeder links with larger bandwidth between satellites and ESs. However, propagation at the Ka band is subjected to rain attenuation and various atmospheric fading mechanisms, which severely reduce the maximum link capacity. As a result, the insufficient capacity of feed link becomes the throughput bottleneck of satellite networks. In order to increase network throughput, it is important to fully use feeder link resources. In this paper, we propose a cooperation scheme to route packets to ESs through a well-resourced feeder link, such that the bandwidth of the feeder links can be fully utilized and the throughput of data downloading at the ESs is maximized. Firstly, we model the satellite network system and the feeder link based on MIMO technology. Then, a Maximum-Flow-Minimum-Cost (MCMF) routing algorithm consisting of two Linear Programs (LPs) is presented to compute maximum-flow routings for data download. Eventually, a variety of simulations are conducted to assess the proposed scheme, which shows that the cooperative routing scheme outperforms the existing SiRRS method in terms of throughput and delay.

## 1. Introduction

**Figure 1:**
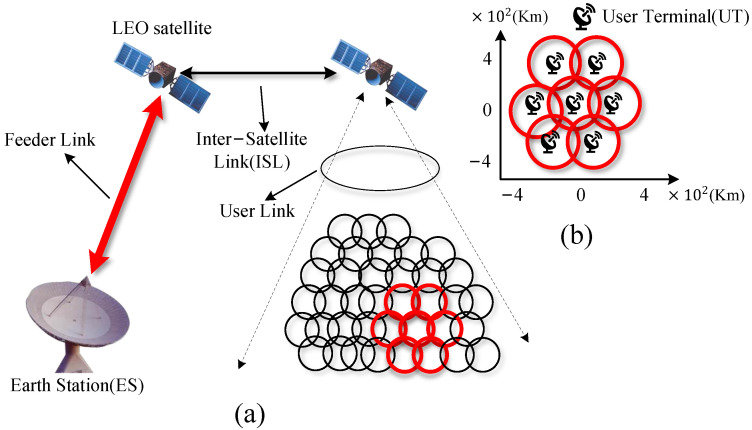
(**a**) Geometrical representation of a LEO satellites network system. (**b**) Schematic diagram of beam coverage.

Due to the Internet’s widespread use and the rocket launch and satellite mass production technology, the LEO satellite network has grown to be a significant component of the mobile communication network. Recently, the Third Generation Partnership Project (3GPP) standards group is working on the convergence of terrestrial mobile communication network and satellite networks to support fifth generation (5G) networks [1]. Currently, many companies have put forward their own plans to construct LEO satellite constellations. For example, SpaceX has launched more than 3000 satellites to provide Internet service to people on the ground. Among them, the latest batch of satellites are equipped with laser terminals to establish inter-satellite links (ISLs) with neighboring satellites. The system architecture of a LEO satellite network system, as shown in Figure 1a, consists of the earth stations (ESs), the satellite network, and the user terminals(UTs). Satellites that can establish links with ESs are called visible satellites and the communication links connecting ESs to their visible satellites are called feeder links. One critical function of satellites is to transfer data from satellite networks to ground core network through earth stations (ESs).

Compared with the traditional SISO(single-input single-output), MIMO communication technology has the advantages of higher transmission rate, spectrum utilization, and power efficiency [2]. Recently, many researchers have proposed to use MIMO technology at Ka-band to build a high-speed feeder link between satellite and ES [3]. Nevertheless, radio propagation at ka-band suffers from atmospheric attenuation mechanisms in the troposphere, such as rain absorption and scattering. Rain attenuation is a phenomenon relative to the rate and frequency of rainfall that can lead to increased path losses, decreasing coverage areas and thus deteriorating system performance. Consequently, the capacity of feeder links in different channel environments will be different. This will lead to inadequate utilization of feeder link bandwidth. For instance, some feeder links may be severely affected by regional rain attenuation and have their capacity reduced, while the the adjacent satellite feeder links still have spare/available bandwidth.

In this paper, we first analyze the capacity of feeder links at Ka-band. Then, we propose the Cooperative Data Downloading (CDD) scheme, in which the traffic converges to the satellites with feeder link through the ISLs. More exactly, the ISLs are used to assist in the data download process by scheduling packets between different feeder links, thus achieving better load balancing. A MFMC routing algorithm is presented to calculate routes for traffic demands. Two linear programs (LPs) are required by the routing algorithm, the first LP finds a maximum flow between the visible satellites, and the second LP minimizes the routing cost given a fixed maximum flow. The above process is repeated until all capacities of feeder links are completely utilized, or until all data from visible satellites can be downloaded to the ESs. In this case, the increased packet delay or packet loss rate caused by a long queue of data in those feeder links with insufficient bandwidth can be alleviated.

At present, many routing algorithms have been proposed to solve the problem of data transmission on LEO satellite networks. In 2001, E. Ekici et al. depicted the network structure of the satellite as a virtual logical topology [4]. By introducing virtual topology, the high dynamic of satellite network can be masked when calculating the route, so that the lightweight goal of routing algorithm can be achieved. Using this work as a foundation, Q. Chen improved the traditional method and proposed a better link connection strategy between satellites to resolve the asynchronous switches caused by the phase difference between satellite orbit planes [5]. Afterwards, J. Li etc. proposed a temporary netgrid model to describe the topology of LEO satellite networks, and utilized the shortest path calculation between grids instead of the shortest path calculation between satellite nodes to make the routing algorithm more efficient [6]. Furthermore, aiming at the challenges of routing protocol convergence and network maintenance in software defined satellite networks, an elastic routing framework is proposed to establish a stable forwarding path for signaling in Ref. [7]. To find the best nexthop for forwarding a packet in the LEO satellite network, Beatriz Soret et al. proposed a new routing protocol named GomHop in which the packet is forwarded to the target orbit as soon as possible [8]. Nevertheless, none of these studies have ever taken into account the feeder links’ constrained capacity, which is a significant bottleneck for the throughput of the LEO constellation networks. In order to increase system throughput, we investigate in this paper how to employ the ISLs to fully use the feeder link.

To increase the throughput of the LEO satellite network, feeder link resources must be utilized effectively. In 1996, Gooley et al. first modeled the Satellite Range Scheduling Problem (SRSP) for time window allocation between satellites and ground stations, when satellites need to transmit data to the ground, and developed an automated scheduling module using mixed-integer programming [9]. In 2004, L. Barbulescu et al. independently proposed the Single-Resource Range Scheduling (SiRRS) based method and the Multi-Resource Range Scheduling (MuRRS) based method after thoroughly examining the SRSP problem [10]. ISLs-based methods for downloading data to ESs have been proposed in recent years. In Ref. [11], Lv Tao et al. considered that the traditional satellite scheduling algorithms have very limited improvement on the system throughput due to the inconsistency between the amount of data to be downloaded and the contact time of the satellite. Therefore, a data downloading scheme based on ISLs is proposed to make the satellite download more data during the contact time. On this basis, X. Jia et al. proposed an iterative optimization strategy to calculate the data transmission demand between satellites according to the length of contact time of each satellite, and modeled the data offloading between satellites as a bipartite graph maximum matching problem in Ref. [12]. In Ref. [13], M. Zhang et al. focused on the energy constraints on satellite data transmission, and modeled the satellite network topology as a transmission graph that varies according to the power level of ISLs. An efficient data offloading algorithm was proposed based on the transmission graph in their research. Nevertheless, the majority of these studies are principally concerned with offloading data for remote sensing observations and cannot be adapted to LEO satellite network broadband communication due to a variety of constraints, including latency, priority, bandwidth, etc.

In this paper, we present the Cooperative Data Downloading (CDD) routing scheme to overcome the problem that a high quantity of user data must be downloaded and feeder link resources are not balanced in a large-scale broadband LEO satellite. Following is the primary creative contribution of this paper:

1. As far as we know, we are the first to use ISLs to facilitate data download in the large-scale broadband LEO satellite network; In order to increase system throughput, traffic is sent through ISLs to satellites with substantial feeder link resources in our scheme;
2. Secondly, we model the topology of LEO satellites network and analyze the data transmission requirements between visible satellites;
3. Finally, we present a maximum-flow minimum-cost routing algorithm to calculate routes for traffic demands, and implemented our algorithm on satellite router.

The remainder of the paper is structured as follows: In Section 2, the system model is presented along with the optimization target of maximizing feeder link bandwidth usage. In Section 3, we describe the ISLs-based collaborative data download scheme. In Section 4, we conduct a simulation experiment and analyze the numerical results. Finally, the paper is summarized in Section 5.

## 2. System Model and Problem Formulation

In this paper, we mainly consider the data downloading problem in a polar orbit constellation. We consider the constellation with *N* orbits, where each orbit contains *M* satellites in the space segment. The angle between the orbital planes is π/N. The LEO satellite network is modeled as a undirected weighted graph, where V is the set of satellites and E is the set of wireless links, including ISLs and feeder links. Let $l_{i,j}\in E$ denote the ISL established by satellites $v_{i}$ and $v_{j}$. Intra-orbit ISLs can always be maintained, however inter-orbit ISLs in the earth’s polar area will be momentarily disconnected when a satellite travels through the region and will be re-established after exiting the zone. There is an orbital seam between neighboring orbits because the satellites in these two orbits are moving in opposite directions. The relative speed of the satellites on both sides of the orbital seam is too fast, which leads to the high cost of link establishment [14], hence this paper assumes that satellites on either side of the orbital seams do not establish.

**Figure 2:**
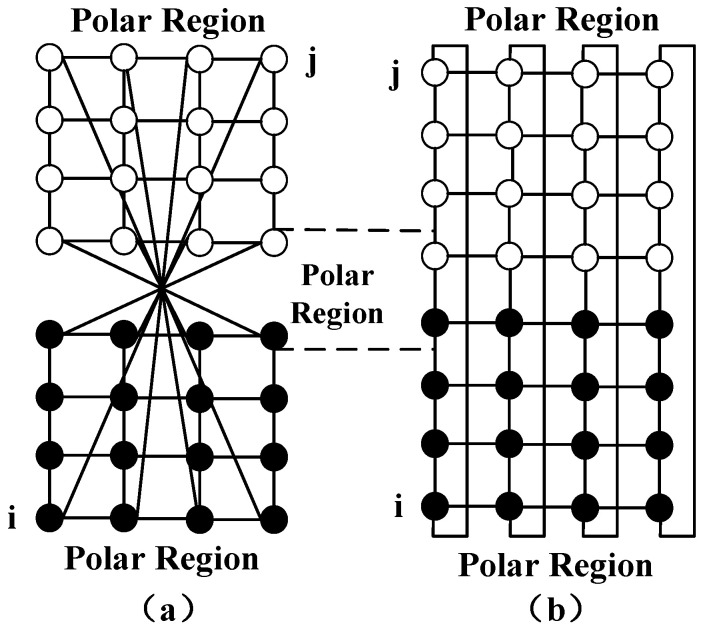
(**a**) Topology diagram of LEO satellites network. (**b**) Topology diagram of LEO satellites network with the upper half rotated 180°. Satellite i is still in its original position on the topology map, and the position of satellite j is changed on the topology map, but the topological connection relationship does not change.

Furthermore, two satellites in neighboring orbits will swap relative positions after traveling over the polar areas, as shown in Figure 2a. When the upper part of Figure 2a is rotated 180 degrees, the topology of the network translates to Figure 2b. In this way, the topology of the LEO satellite network can be modeled as a set two-dimensional mash structure. The dynamic nature of a network topology is principally driven by the establishment of inter-orbit ISLs, as satellites enter and exit the polar area, and by the interference that prevents laser terminals from establishing ISLs due to a high bit error rate.

Let $V_{ES}\in V$ represent the set of visible satellites that are located above the ESs and can establish feeder links with the ESs. When the satellite $v_{i}$ can establish the feeder link $W_{i}$ with the ground station, the satellite will join the set *V*. When the feeder link of the satellite is disconnected, it will exit the set *V*. There are many ESs scattered around the globe. Each ESs is equipped with *k* MIMO antenna arrays, each of which can establish a feeder link to a visible satellite. Due to the fact that all satellites follow their own orbits, their contact time windows with ESs (i.e., the start and finish times of windows) are known. On account of that the satellite travels with period *T*, the satellite network topology is likewise regularly transformed with period *T*. This paper separates the satellite operating period into time slots of the same size and duration of τ, such that the timeline can be represented as 0, 1τ, 2τ, …, *T*. The network topology in each time slice is fixed so as to eliminate the influence of satellite network dynamics on routing algorithm.

### 2.1. Feeder Link Model and Capacity

Compared with the traditional SISO, MIMO communication technology has the advantages of higher transmission rate, spectrum utilization, and power efficiency. Recently, many scholars proposed to use MIMO technology at Ka-band to construct high-speed feeder link between satellites and ESs. However, ka-band propagation is influenced by atmospheric attenuation mechanisms from the troposphere, which will result in a difference in feeder link capacity. In this section, we elaborate MIMO channel model at Ka-band and the impact of rain attenuation on feeder link capacity.

**Figure 3:**
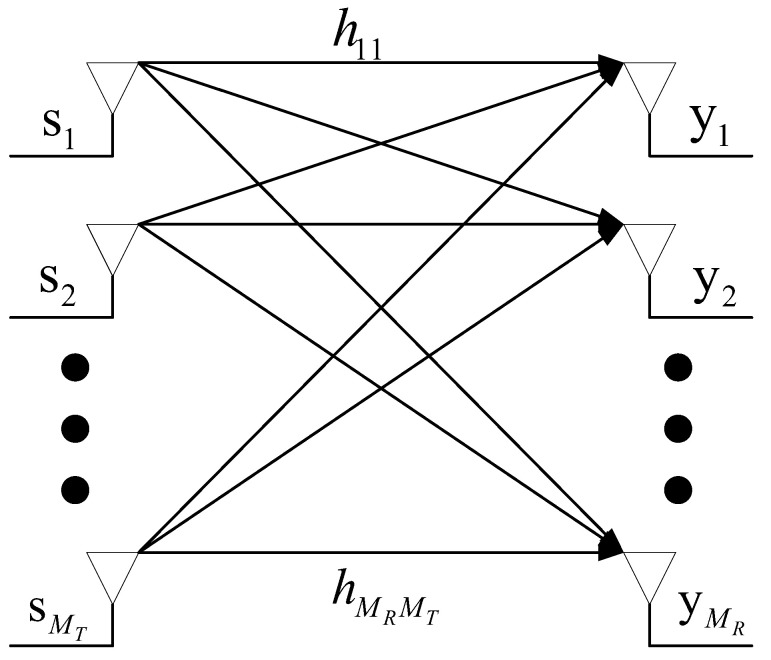
MIMO system.

As shown in Figure 3, a MIMO system can be composed of $M_{T}$ receiving antennas and $M_{R}$ transmitting antennas between the satellite and the ES. Let H denote the channel matrix between the satellite and the ES. The input-output relation in a MIMO channel for constructing the feeder link can be expressed as [15]:

(1) $$y\left[k\right]=\sqrt{\frac{E_{S}}{M_{T}}}Hs\left[k\right]+n\left[k\right],$$

where $E_{S}$ is the average energy of the symbols. n[k] is the $M_{R}\times 1$ noise vector of independent ZMCSCG random variables, s[k] is the $M_{T}\times 1$ vector of signals transmitted from each antenna and y[k] is the $M_{R}\times 1$ vector of signals received by each antenna.

Some studies researched by other scholars have led to the “log-det” capacity formula in Ref. [16]. In this paper, the channel capacity of a MIMO channel can be obtained from the channel matrix:

(2) $$C=\max_{Tr\left(Q\right)=M_{T}}\left\{\log_{2}det(I_{M_{R}}+\frac{E_{S}}{M_{T}N_{0}}\mathrm{HQ}H^{H})\right\}.$$

In (2), $Q=E[ss^{H}]$ is the covariance matrix of the transmitted signal vector *s* and $I_{m}$ represent the m×m identity matrix. Once the distribution of *H* is known, the maximum data rate *C* that the channel can transmit can be calculated. The element $h_{i,j}$ of H denotes the channel parameter from the *j*th transmitting antenna to the *i*th receiving antenna. The propagation and scattering impacts are considered in this paper. For convenience, $h_{i,j}$ is written as [17].

(3) $$h_{i,j}=h_{1\underline{\;}i,j}h_{2\underline{\;}i,j}$$

where $h_{1\underline{\;}i,j}$ denotes path loss and $h_{2\underline{\;}i,j}$ is a complex random variable, which denotes the fading coefficient induced by random multipath propagation or the incoherent scattering in the troposphere. When the signal of satellite feeder link passes through the atmosphere, it will be absorbed and scattered by oxygen molecules and water vapor condensates in the troposphere, resulting in atmospheric loss. The extreme rain attenuation will reduce feeder link channel capacity and cause bandwidth imbalance of feeder links.

### 2.2. Problem Formulation

In the scenario studied in this paper, each visible satellite will receive data, which needs to be downloaded to the ES, denoted by $b_{i}$. Given the download data rate of each visible satellite, a set of satellites and an ES, our challenge is to design a strategy that allows visible satellites to utilize ISLs to transfer data from satellites with high data loads to those with light loads, so that the bandwidth resources of feeder links can be fully used and the LEO system’s download throughput is maximized. Let $B_{i}$ denotes the actual capacity of the feeder link of satellite $v_{i}$, and it also denotes the maximum data rate that can be downloaded to the ES through this feeder link. The objective of our optimization is to minimize the packet loss rate due to feeder link congestion, which can be given by:

(4) $$\min\Sigma_{i}\left|b_{i}-B_{i}\right|,$$

where

(5) $$∥b_{i}-B_{i}|=\left\{\begin{array}{cc} b_{i}-B_{i} & when\;b_{i}-B_{i}>0\\ 0 & when\;b_{i}-B_{i}<0. \end{array}\right.$$

For simplicity, the following assumptions are made:

1. The size of data packets delivered across all satellites is the same. Under this assumption, the amount of data can be expressed in terms of the number of packets;
2. Each satellite is equipped with four laser terminals to establish ISLs with four adjacent satellites in the same orbit and different orbits, and a wireless signal terminal to establish a feeder link with ESs. These links work in simplex mode.

## 3. The Proposed Downloading Scheme

### 3.1. Overview of Our Solution

Our scheme for maximizing the satellite system throughput of downloading data is to transmit data between visible satellites via ISLs, so as to reduce the load of feeder links with insufficient bandwidth. In order to allocate the feeder link bandwidth resources, we must determine the amount of data rate this satellite can receive from others or need to transfer to others. To get the traffic demands, we calculate the sender and receiver of inter-satellite transmission. Then, a constrained MCMF routing algorithm is described to compute maximum-flow routing for all traffic demand needs inside a network’s capacity area subject to routing cost limitations. Two linear programs (LPs) are required by the routing algorithm. The first LP finds a maximum flow between the visible satellites, and the second LP minimizes the routing cost given a fixed maximum flow. After one round of calculations, some feeder lines may have capacity surpluses, but others may have data waiting to be downloaded. Therefore, the above process needs to be iterated until all data from visible satellites can be downloaded to the ES or all feeder links have no spare capacity.

### 3.2. Calculating the Source and Sink of Intersatellite Transmission

To utilize the ISLs to aid the feeder link in downloading data, it is important to first define the traffic transmission requirements, mainly including the source satellite, destination satellite, and the rate of data transmission. Then, we can calculate the route between visible satellites according to these requirements.

The set of real numbers is denoted by R. Let $D\in R^{N\times N}$ represent the traffic demand matrix, where each element represents the required data transmission rate of two visible satellites. At the beginning of each time slice nτ, each satellite updates the capacity and data transmission rate of each link, and the topology of the network changes along with it.

When $b_{i}-B_{i}<0$, the satellite $v_{i}$ can receive additional download data stream. Let $l_{i}$ denote the rate of this stream and let $V_{i}$ denote the set of such satellites. On the basis of the capacity of this satellite’s ISLs and feeder link, $l_{i}$ can be calculated as follows:

(6) $$l_{I}=\min\left\{B_{i}-b_{i},\Sigma \left(L_{ji}-l_{ji}\right)\right\}.$$

where $L_{ji}$ denotes the input ISLs capacity of node $v_{i}$ and $l_{ji}$ denotes the data transmission rate of the link $l_{j,i}$.

When $b_{i}-B_{i}>0$, satellite $v_{i}$ needs to transfer data stream $l_{O}$ to other visible satellites. Let $l_{O}$ denote the rate of this stream and let $V_{O}$ denote the set of such satellites. On the basis of the capacity of this satellite’s ISLs and feeder link, $l_{O}$ can be calculated as follows:

(7) $$l_{O}=\min\left\{b_{i}-B_{i},\Sigma \left(L_{ij}-l_{ij}\right)\right\}.$$

where $L_{ij}$ denotes the output ISLs capacity of node $v_{i}$ and $l_{ij}$ denotes the data transmission rate of the link $l_{i,j}$.

We arrange the satellites in descending order based on the $l_{O}$ and $l_{I}$ of each satellite in the $V_{O}$ and $V_{I}$ set. The $\min(l_{O},l_{I})$ between the two satellites is utilized to calculate the element of the source-to-destination demand flow rate D(i,j). Consequently, matrices *D* of the traffic demand are obtained.

### 3.3. The Minimum-Cost Maximum-Flow Algorithm

**Figure 4:**
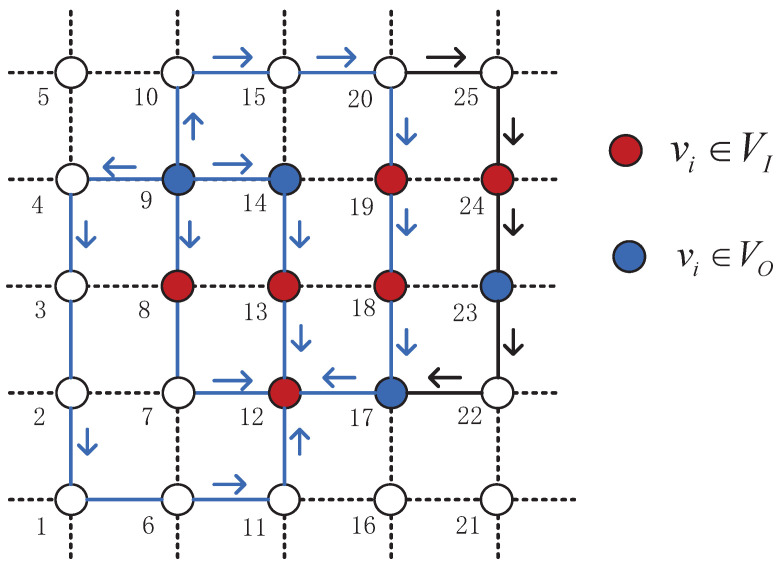
Multi-hop routing in a LEO satellite network. The number next to the node represents the ID of the satellite.

This section will introduce the MCMF routing algorithm. As shown in Figure 4, the two minimum cost paths between (9, 12) are P(9,8,7,12) and P(9,14,13,12), which are identified by the blue arrows and lines. The maximum flow allowed between (9, 12) in Figure 4 includes four paths, and the other maximum flow paths with larger costs are identified by black arrows and lines. The traditional multi-data stream maximum flow linear programming problem [18] has the following three problems: 1. The possibility of data transmission on a shorter or longer path is the same, so the transmission cost cannot be minimized; 2. Because the transmission cost cannot be minimized, it may cause loopback; 3. On-board computing resources are limited, it is also difficult to solve the LPs problem in a timely manner even if in a limited network size.

In order to solve the above-mentioned problems, the distance bandwidth product (BD) is proposed as the link cost, allowing shorter paths to be planned for larger traffic, minimizing transmission costs, and preventing loopback. Secondly, each data stream’s computation is constrained by subgraph restrictions depending on energy and computational resources, thereby greatly reducing the computational complexity. Once a subgraph $G^{c}$ is generated for every flow c∈C, the Constrained MCMF algorithm including two LPs can be formulated.

### 3.4. The Maximum-Flow LP and the Minimum-Cost LP

In the satellite network topology map G(V,E), we need to calculate the distance-constrained subgraph $G^{c}\left(V^{c},E^{c}\right)\in G\left(V,E\right)$ through the traffic demand matrix $D\in R^{N\times N}$. First, the Dijkstra algorithm is used to compute the shortest path from all satellites to other satellites in the visible area of an ES. Then, a set of candidate links and nodes is initialized to NULL. Let N(s,d) denote the number of hops in the shortest path between the pair of source and destination (s,d). After that, the algorithm traverses other intermediate nodes in the area, using M(s,d) to represent the number of hops of the shortest path of (s,d), and an appropriate hop threshold HT can be taken based on the computing resources of the network. If the satellite *v* satisfies the inequality N(s,v)+N(v,d)≤N(s,d)+HT, the satellite *v* and its four ISLs as the element of the subgraph node set $V^{c}$ and edge set $E^{c}$. Once the subgraph for each data flow has been calculated, two linear programmings can be formulated to the MCMF algorithm.

By constraining the data flow distance between each pair of source/destination (s,d), we can obtain the matching subgraph. The linear programming issue of the greatest data flow of multiple sources and sinks constrained by the subgraph (LP#1) is represented by Equation (8).



(8)
$$\max:l^{*}=\sum_{c\in C}l_{in}^{c}\left(d_{c}\right)$$


**Subject to:**


(8a)0≤lc(e)∀c∈C,∀e∈Ec(8b)lc(e)≤L(e)∀c∈C,∀e∈Ec(8c)∑c∈Clc(e)≤L(e)∀c∈C,∀e∈E(8d)linc(v)=loutc(v)+bc(v)∀c∈C,∀v∈Vc(8e)lincsc=0∀c∈C(8f)loutcdc=0∀c∈C(8g)loutcsc≤Dc∀c∈C



$$l^{*}=\sum_{c\in C}l_{in}^{c}\left(d_{c}\right).$$



   Let $l^{c}\left(e\right)$ denote the traffic rate of commodity *c* on link *e*. Let $l_{in}^{c}\left(v\right)$, $l_{out}^{c}\left(v\right)$ and $b^{c}\left(v\right)$ denote the in/out overall traffic rate of ISLs and the traffic rate of the feeder link in satellite *v*, respectively. Let $D^{c}$ denote the request flow *c* of the matrix *D*. Equation (8a)–(8c) require that the sum of all commodity traffic rates on a link e is less than the link capacity L(e). Equation (8d)–(8f) are limited to the subgraphs for each commodity flow. Equation (8g) ensures the receive rate of each commodity at most $D^{c}$. The maximum flow of multiple source-destination in the network can be attained by solving this LP.

To minimum the routing cost, the second linear programming problem (LP#2) is represented by Equation (9).



(9)
$$\min:y^{*}$$


**Subject to:**


(9a)routcsc=Γc∀c∈C(9b)∑c∈Crc(e)≤Z(e)∀c∈C,∀e∈E(9c)rinc(v)=routc(v)∀c∈C,∀v∈V′(9d)rincsc=0∀c∈C(9e)routcdc=0∀c∈C



$$y^{*}=\sum_{c\in C}\sum_{e\in E}x_{e}^{c}\times M\left(e\right).$$



Let $\Gamma^{c}$ denote the maximum flow of the commodity *c* obtained by solving the maximum flow LP, and $V^{\prime }=V-\left(s_{c},d_{c}\right)$. In the above formula, Equation (9a) requires the fixed rate of each commodity flow obtained by LP#1. The remaining resemble the preceding constraints. The objective of our function is to minimize the sum of flow rates $x_{e}^{c}$ multiplied by link cost M(e). In our scheme, the greedy approach is utilized to solve these two LPs in order to achieve the maximum flow and minimum cost routing.

### 3.5. Iterating the Algorithm

**Figure 5:**
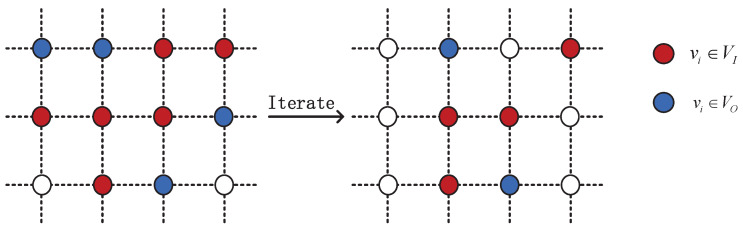
Schematic diagram of the network iteration.

After finishing a round of computations in the preceding sections, it is necessary to update and iterate the network topology because the link status changes, as shown in Figure 5. In order to reduce the iteration rounds and speed up the convergence speed, if the satellites in the $V_{I}$ set $l_{I}<\alpha$, then the satellites exit the set $V_{I}$, changing from a red node to a white node. If the satellites in the $V_{O}$ set $l_{O}=0$, they will also exit the set $V_{O}$, changing from a blue node to a white node. The rest of the nodes of set $V_{I}$ and $V_{O}$ repeat the preceding calculation procedure until the number of nodes in set $V_{O}$ or $V_{I}$ is NULL, that means, the feeder resources are completely filled and the throughput reaches its maximum. The algorithm terminates the iterative process until the next time slot τ, at which point it will begin again.

The flow chart of this algorithm is shown in Algorithm 1.
**Algorithm 1:** Downloading Algorithm
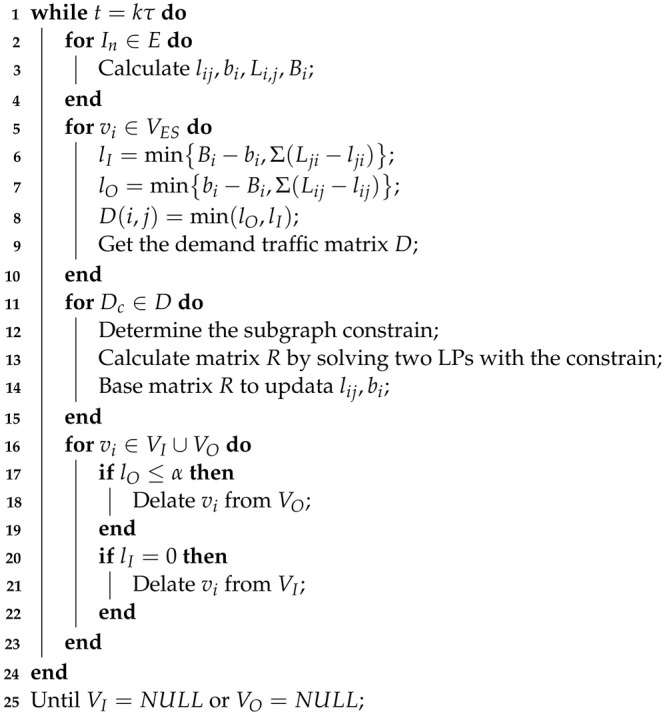


## 4. Discussion

In order to obtain the simulation performance of our proposed Cooperative Data Downloading (CDD) routing scheme, we used the Satellite Tool Kit (STK) to construct the satellite network scene and jointly carried out algorithm simulation with OPNET modeler and MATLAB.

### 4.1. Satellite Constellation in Simulations

In the simulation scenario, we chose a representative Walker delta, Globalstar constellation. The constellation has 8 orbits with 6 satellites in each orbit, and the angular distance between the planes is 60°. The orbital period is 113 min, and the orbital inclination is 52°. Globalstar satellites are equipped with four laser terminals with a bandwidth of 10GHz for ISL communication, and a 1GHz-bandwidth wireless terminal for communicating with ES, as shown in Table 1. The simulation starts at 8:00:00 UTC on 15 August 2022, and stops at 9:54:00 UTC on 15 August 2022. ES is located in Shanghai, 122.12° longitude and 31.53° latitude.

### 4.2. Simulation Scenario Construction

**Figure 6:**
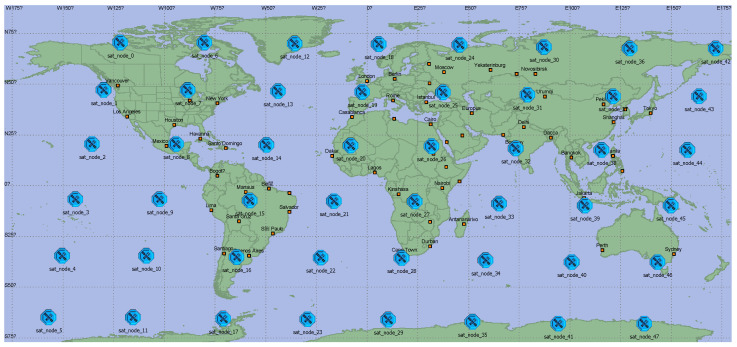
The scenario of the simulation.

**Figure 7:**
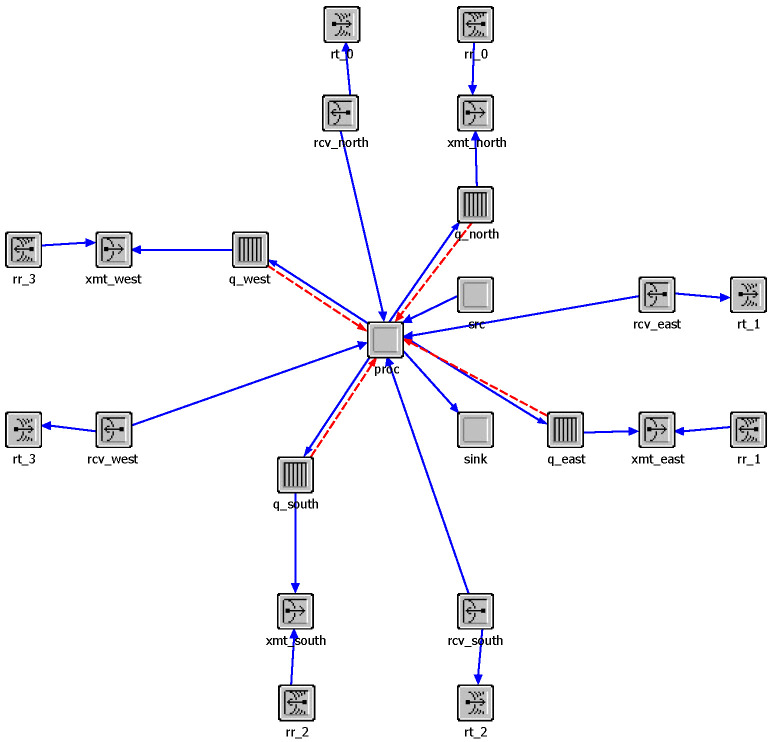
Satellite model.

OPNET modeler was used to build our simulation system. First, we import satellite orbit parameters from STK to construct the network scenario model, as shown in Figure 6. After that, we build the node model of the satellite, as shown in Figure 7. The src module is used as the data source to simulate the flow distribution of the user terminal. The proc module serves as the CPU of the satellite and is used to build the network protocol and the algorithm proposed in this paper. The four signal receiving and transmitting terminals serve as laser terminals to establish ISLs with adjacent satellites.

**Figure 8:**
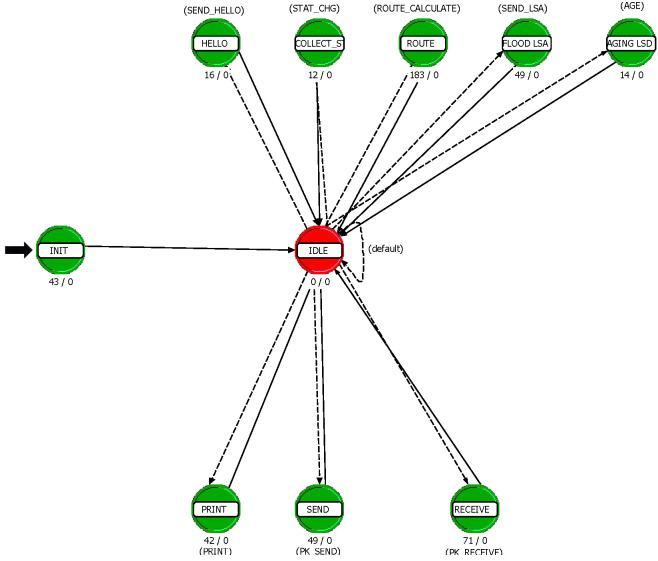
Process model.

In the proc module, we use the finite state mechanism to build the network protocol and algorithm, as shown in the Figure 8. The main functions of proc module include initialization, packet sending and receiving processing, signaling generation and broadcasting, and routing algorithm implementation.

### 4.3. Simulation Results and Analysis

We assume that the data received by the satellite network follows a normal distribution [12]. The mean value of the normal distribution μ represents the average quantity of data received from users. The variance σ of the distribution is set to range from 0.05μ to 0.5μ. We compare our scheme with the method named SiRRS (Single-Resource Range Scheduling) [9], which does not utilize ISLs to transfer data between satellites.

Changing the mean value μ of the normal distribution allows us to simulate the performance of the scheme under two distinct data download load scenarios. By light and heavy load, the total amount of data to be downloaded is set to around 40–50% and 80–90% respectively, of the capacity of the ES. Throughput and end-to-end delay are selected as measures of scheme performance. The throughput is defined as the proportion of actual downloaded traffic to overall network traffic. End-to-end latency is defined as the time between when a packet is sent to the network and when it is received by an ES.

**Figure 9:**
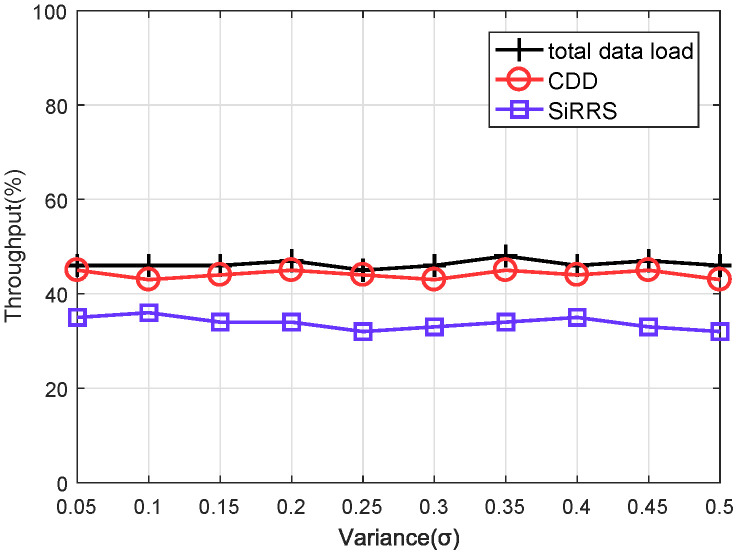
Throughput of light data load.

**Figure 10:**
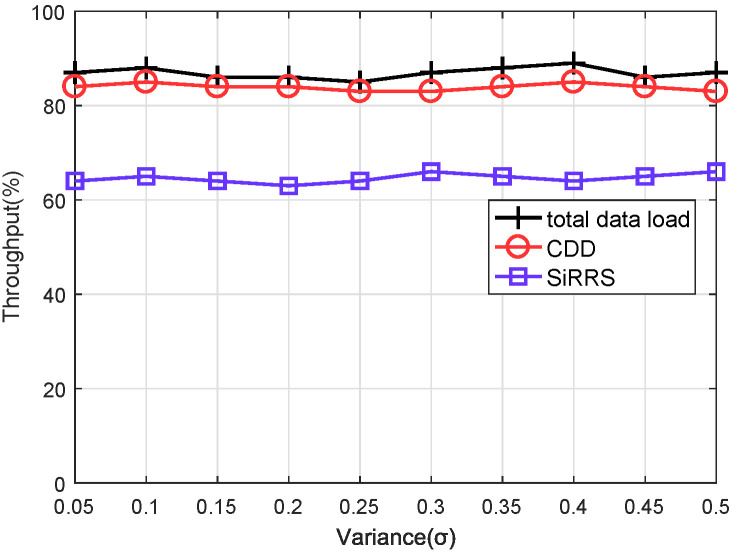
Throughput of heavy data load.

**Figure 11:**
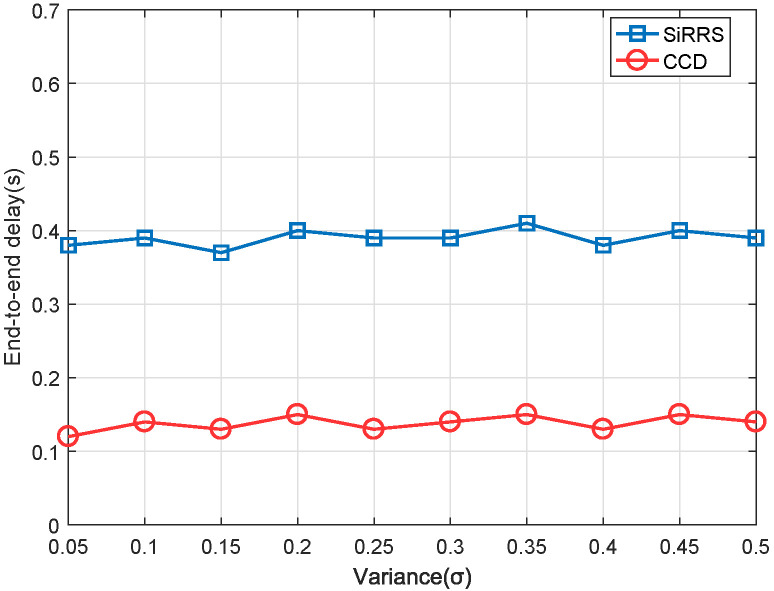
End-to-end delay of light data load.

**Figure 12:**
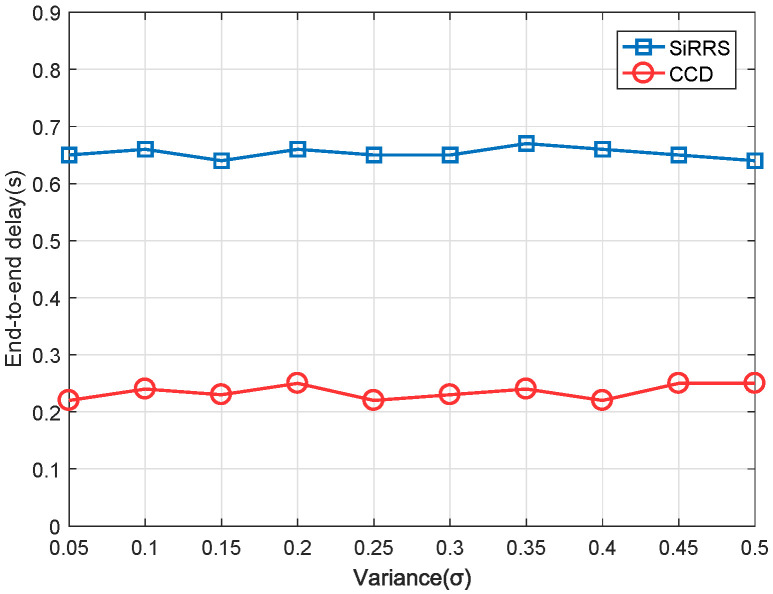
End-to-end delay of heavy data load.

Figure 9 and Figure 10 are the simulation results of throughput under the light load and heavy load, respectively, and Figure 11 and Figure 12 are the simulation results of end-to-end delay under the light load and heavy load, respectively. The total data load in the figures represents the ratio between the total quantity of data received by all satellites and the capacity of the ES. We can draw the following conclusions from Figure 9, Figure 10, Figure 11 and Figure 12.

(1) From Figure 9 and Figure 10, it is obvious that our proposed scheme (CDD) outperforms the SIRRS method in terms of throughput. The throughput performance of the CDD scheme remains close to the network load with the change of the data load. In this way, visible satellites can download almost all user data. However, in the simulation using the SIRRS scheme, the system throughput starts to decrease when the network load is heavy. The reason for this phenomenon is when the capacity of feeder links is reduced due to rain attenuation, a large amount of data still needs to be downloaded to ES through these feeder links. When the waiting time of these data in the satellite cache queue exceeds the lifetime, they will be discarded and the system throughput will decrease. The CDD scheme avoids the above situation and makes it perform better than the method that does not use ISL data transfer.

(2) From Figure 11 and Figure 12, our proposed scheme also outperforms the SiRRS method in terms of end-to-end delay. The end-to-end delay obtained by the simulation of this scheme is still about 0.25 s when the network load is very heavy (80–90%). The average delay of SiRRS method is 0.65 s, which means that many packets will become clogged in the cache for longer than their lifetime.

**Figure 13:**
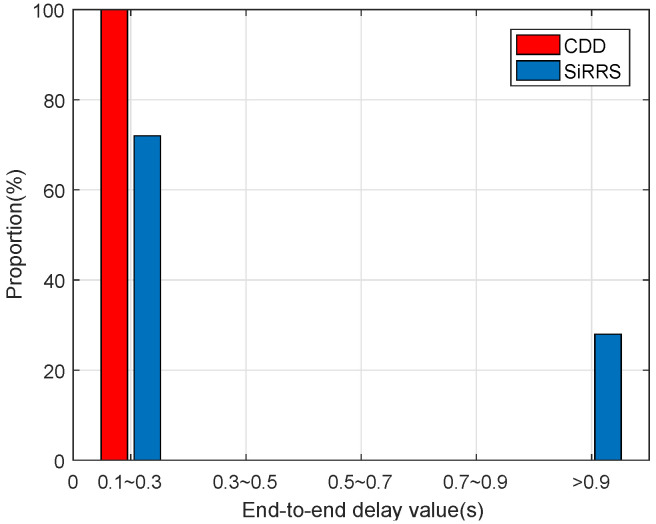
Distribution of delay of light data load.

**Figure 14:**
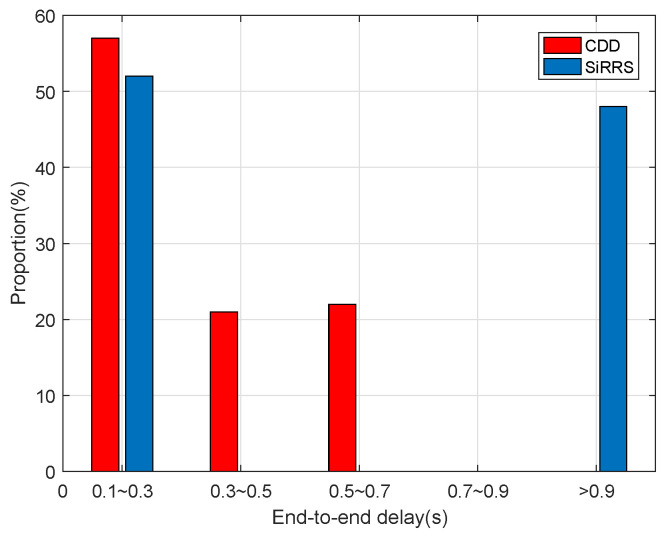
Distribution of delay of heavy data load.

Figure 13 and Figure 14 show the data delay distribution separately under different data loads. As can be seen from Figure 14, when the network load is very heavy, nearly half of the packet delay in the simulation results obtained by SIRRS method is more than 0.9 s, while the packet delay of CDD scheme is all less than 0.7 s. The cause of this phenomenon is that a large number of packets accumulate on feeder links with low capacity, resulting in a dramatic increase in queuing delay in SiRRS method, while the CDD scheme reduces the end-to-end delay of packets by transferring the data to satellites with abundant feeder link capacity.

## 5. Conclusions

In this paper we proposed a cooperative routing algorithm that allows visible satellites to transfer data among themselves by using the ISLs when some feeder links are congested, such that high packet loss rates caused by long queuing delay can be avoided and to maximize the data downloading throughput. A Maximum-Flow-Minimum-Cost (MCMF) routing algorithm is presented to compute maximum-flow routings for data download in CDD scheme. By using STK, OPNET modeler, and MATLAB, extensive simulations have been executed to test our proposed approach. The simulation results demonstrate that our proposed CDD scheme is superior to the method that does not use the ISL data transfer in terms of throughput and delay.

## Figures and Tables

**Table 1:** Parameters of the constellation.

Parameter	Value
The number of orbits	8
The number of satellites per orbit	6
The orbital period	113 min
The inclination of orbits	52°
The angle between adjacent orbits	60°
The bandwidth of ISLs	10 G
The bandwidth of feeder links	1 G

## Data Availability

The data presented in this study are available on request from the corresponding author.
